# *TNF-alfa* Gene Polymorphism Associations with Multiple Sclerosis

**DOI:** 10.3390/jcm13133693

**Published:** 2024-06-25

**Authors:** Lukas Kalvaitis, Greta Gedvilaite-Vaicechauskiene, Loresa Kriauciuniene, Renata Balnyte, Rasa Liutkeviciene

**Affiliations:** 1 Medical Faculty, Lithuanian University of Health Sciences, Medical Academy, LT-50161 Kaunas, Lithuania; lukas.kalvaitis@stud.lsmuni.lt; 2 Neuroscience Institute, Lithuanian University of Health Sciences, Medical Academy, LT-50161 Kaunas, Lithuania; loresa.kriauciuniene@lsmuni.lt (L.K.); rasa.liutkeviciene@lsmuni.lt (R.L.); 3 Department of Neurology, Lithuanian University of Health Sciences, Medical Academy, LT-50161 Kaunas, Lithuania; renata.balnyte@lsmuni.lt

**Keywords:** multiple sclerosis, gene polymorphism, *TNF-alpha*

## Abstract

**Background:** *TNF-α* has a dual role in multiple sclerosis (MS), contributing to both protective and harmful effects. It activates immune cells, promotes the formation of inflammatory lesions in the central nervous system, and stimulates the production of other pro-inflammatory cytokines and chemokines, leading to myelin destruction and neuronal damage. Our research focused on investigating the relationship between *TNF-alpha* (rs1800630, rs1800629, and rs361525) gene polymorphisms and MS. **Methods:** 250 healthy controls and 250 multiple sclerosis (MS) patients were included in the study. DNA was extracted from leucocytes from peripheral venous blood by salt precipitation. Single nucleotide polymorphisms (SNPs) were tested using RT–PCR. Statistical analysis of the data was performed using IBM SPSS Statistics 29.0 data analysis software. **Results:** The analysis revealed that the rs361525 AG genotype was significantly less frequent in the MS group compared to the control group (4.0% vs. 7.2%, *p* = 0.042). Sex-specific analysis showed a significant difference in genotype distribution (GG, AG, AA) among males between the MS group and the control group (97.7%, 0%, 2.3% vs. 90.6%, 9.4%, 0%, *p* = 0.005). For the rs1800629 polymorphism, significant results were also found. In subjects younger than 39 years, the A allele was significantly less frequent in the MS group than in the control group (8.6% vs. 15.0%, *p* = 0.030). The most robust model indicated that the AA genotype reduced the odds of MS by approximately 2 fold compared to the AG + GG genotype (*p* = 0.044), and each A allele reduced the odds of MS by approximately 2 fold (*p* = 0.028). The rs1800630 A allele was significantly more common in males in the MS group than in the control group (21.0% vs. 12.9%, *p* = 0.046). **Conclusions:** In conclusion, our study identifies significant associations between *TNF-alpha* gene variants and MS. Specifically, the rs631525 AG genotype was less common in the MS group, with notable sex-specific differences observed. The rs1800629 A allele was statistically significantly less frequent in the MS group than in the control group, and the AA genotype reduced the odds of MS occurrence by ~2 fold compared with the AG + GG genotypes. Additionally, each A allele of rs1800629 was linked to a 2-fold decreased odds of MS occurrence. In males, the rs1800630 A allele was more frequent in the MS group. These findings highlight the relevance of *TNF-alpha* genetic variations in MS susceptibility, suggesting potential avenues for further research and therapeutic exploration.

## 1. Introduction

Multiple sclerosis (MS) is a disease that affects the central nervous system (brain and spinal cord) and can lead to severe physical or cognitive disabilities and other neurological disorders [1,2]. It is considered a chronic inflammatory disease of the central nervous system that occurs when our body’s immune cells mistakenly defend themselves and attack the myelin that covers the nerve cells in the CNS (brain, optic nerves and spinal cord) [3]. These damaged areas, scars and demyelinating plaques form, impairing nerve impulse transmission. Therefore, the myelin sheath and axons are lost over time and with progressive demyelination, damaging the central nervous system [4]. Myelin is a substance that coats the axons of nerve cells and enables them to transmit impulses more efficiently and quickly. It is necessary for normal sensory, cognitive and motor functions, so its damage manifests itself in symptoms such as visual disturbances, bladder and bowel problems as well as movement and coordination disorders; the most common symptom is fatigue [1,4]. MS manifests in different forms, including relapsing–remitting MS (RRMS), which is characterized by clearly defined attacks of worsening neurologic function followed by partial or complete recovery periods (remissions). Secondary progressive MS (SPMS) may follow an initial relapsing–remitting course and is characterized by a progressive worsening of neurologic function over time. Primary progressive MS (PPMS) is characterized by a steady progression of disability from onset, without initial relapses or remissions. The course of MS can be highly variable, with some individuals experiencing mild symptoms and others facing significant physical and cognitive challenges [5]. MS is usually diagnosed in young people between the ages of 20 and 40, but the disease can also occur earlier. This pathological condition affects more women than men (sex ratio 2.5:1) [6,7] and the prevalence varies by geographical area and is around 120 per 100,000 people [8]. There is currently no single accurate test that can be used to diagnose MS as it is a complex, multifactorial disease. The diagnosis is only confirmed after the doctor has carried out a detailed medical history, physical examination and neurological examination [4]. One of the most common methods of monitoring the disease and damage to the nervous system is a magnetic resonance imaging (MRI) scan of the brain and spinal cord to detect changes characteristic of MS. Scar tissue and CNS damage can be detected with this examination. The final diagnosis of MS is made on the basis of the McDonald criteria, named after the neurologist W. Ian McDonald. The patient must have experienced at least one sudden worsening of symptoms, and central nervous system lesions and their evolution over time must have been identified and assessed [9,10].

Inflammatory and immune mechanisms are thought to play the most important role in the pathogenesis of MS, but it is not fully understood whether inflammation is the initial event of the pathophysiological processes or only a secondary response to an unknown pathogen [11]. There are many scientific papers that claim that the immune system has the greatest influence on the manifestation of the disease. According to this theory, MS occurs when autoreactive cells (macrophages, T-killer cells, lymphokines and antibodies) invade the CNS and trigger an autoimmune response against myelin or oligodendrocyte antigens [12]. Other studies suggest that it is a multifactorial disease, meaning that both environmental and genetic factors may play a role in the development of MS [13]. However, the exact cause of this disease is unknown.

In order to clarify the influence of genetic factors on MS, a clustering of twins and families was carried out. The results show that identical twins have a higher clinical concordance rate (25–30%) than fraternal twins (3–7%), possibly indicating the low penetrance of this disease. The risk of developing MS in first-degree relatives is estimated to be 3, which is 3-fold higher than in second- and third-degree relatives, where it is 1. The increased risk of developing MS only exists if both parents are affected [14].

Tumor necrosis factor-alpha (TNF-α) is an inflammatory cytokine produced by macrophages or monocytes during acute inflammation [15]. It is the most important regulator of inflammatory reactions and is also involved in the pathogenesis of some inflammatory and autoimmune diseases [16]. TNF-α is a homotrimeric protein consisting of 157 amino acids and is mainly produced by activated macrophages, T lymphocytes and natural killer cells [17]. TNF-α is known to be responsible for the production of inflammatory molecules, chemokines and cytokines. It can be present in soluble (sTNF-α) and transmembrane (tmTNF-α) forms. The transmembrane form (tmTNF-α) is converted into a soluble form (sTNF-α) by an enzyme (TACE) and then used to regulate biological processes by type 1 (TNFR1) and type 2 (TNFR2) receptors [18]. TNFR1 is expressed in all human tissues and is the main signaling receptor for TNF-α. TNFR2 is normally expressed in immune cells and is responsible for a limited biological response. In general, TNF-α is very important for a normal immune response. TNF-α can activate the regulation of the immune system, but overexpression of TNF-α can be harmful and life-threatening. As TNF-α is not only involved in the development of inflammatory diseases but also autoimmune diseases, TNF-α inhibitors have been developed and are now used to treat autoimmune diseases such as rheumatoid arthritis (RA). Therapeutic drugs act as antagonists, blocking the interaction of TNF-α with TNFR1 and TNFR2 receptors, and in some cases as agonists, stimulating reverse signaling-induced apoptosis of TNF-α-producing immune cells [18,19]. The human *TNF-α* gene is located on chromosome 6 at position p21.3. It consists of four exons and three introns and spans approximately 3 kilobases. The *TNF-α* gene encodes the TNF-alpha protein, a pro-inflammatory cytokine involved in immune system responses and the regulation of inflammation. It is assumed that the *TNF-α* gene is involved in the regulation of inflammatory reactions [20]. Therefore, the single-nucleotide polymorphisms rs1800630, rs1800629 and rs361525 of this gene were selected to search for associations with MS. The *TNF-α* rs1800630 polymorphism is a mutation in which adenine is replaced by cytosine. The rs1800629 polymorphism is a substitution of adenine for guanine, and the rs361525 polymorphism is a mutation from adenine to guanine. Changes in the *TNF-α* gene are associated with altered production of the TNF-α protein and susceptibility to certain diseases [21,22]. One known genetic variant is the single-nucleotide polymorphism (SNP) rs1800629, also known as *TNF-308* A/G. This SNP is located in the promoter region of the *TNF-alpha* gene and is associated with changes in *TNF-α* expression levels. The A allele is associated with increased production of TNF-alpha and is linked to various inflammatory diseases such as rheumatoid arthritis, inflammatory bowel disease and asthma. The TNF-863A/C polymorphism is also associated with altered TNF-α production. The A allele is associated with higher *TNF-α* expression compared to the C allele. Under certain conditions, this increased production of TNF-α can contribute to increased inflammation and dysregulation of the immune system [22]. *TNF-α* plays a complex role in MS and contributes to both protective and detrimental effects. *TNF-α* is involved in the activation of immune cells, the recruitment and formation of inflammatory lesions in the central nervous system. It stimulates the production of other pro-inflammatory cytokines and chemokines, which activates immune cells to migrate to the site of inflammation. This can lead to the destruction of myelin, the protective sheath of nerve fibers, and contribute to neuronal damage [15,23,24].

So, our research aimed to investigate the association of *TNF-alfa* (rs1800630, rs1800629, and rs361525) gene polymorphisms with the predisposition to multiple sclerosis. 

## 2. Methods

### 2.1. Study Design

The case–control study was approved by the Ethics Committee for Biomedical Research of the Lithuanian University of Health Sciences (No. BE-2-/102). It was conducted at the Lithuanian University of Health Sciences, NI, Ophthalmology laboratory and in the Department of Neurology of the Lithuanian University Hospital of Health Sciences (Kaunas, Lithuania). All subjects who participated in this study gave written informed consent. Due to ethical reasons, the data presented in this study are available on request from the corresponding author.

Two groups were randomly formed during this study:

- Group I: patients with multiple sclerosis (*n* = 250).
- Group II: healthy subjects (*n* = 250).

The diagnosis of MS was confirmed using the 2017 McDonald diagnostic criteria, which include clinical symptoms/relapses, MRI findings of the brain and spinal cord showing typical demyelinating lesions (as per MAGNIMS criteria), and the presence of positive oligoclonal bands in cerebrospinal fluid [11,12].

Inclusion Criteria for the MS Group:

- Patients diagnosed with multiple sclerosis, confirmed using the 2017 McDonald diagnostic criteria. This includes the presence of positive oligoclonal bands, typical demyelinating lesions on brain or spinal cord MRI scans (according to MAGNIMS criteria), and clinical symptoms or relapses.
- Males and females aged between 18 and 99 years.

Exclusion Criteria for the MS Group:

- Patients younger than 18 years.
- Patients who have received a transfusion of blood or blood components within the past four weeks.
- Patients who have received treatment with growth factors that influence blood production within the past four weeks.

Patients with other systemic diseases (diabetes mellitus, oncological diseases, systemic tissue disorders, chronic infectious diseases, autoimmune diseases, conditions after organ or tissue transplantation).

The control group consisted of 250 healthy individuals, matched by age and sex to the MS group, who attended LSMUL, KK, the Neurology Clinic, and the Eye Clinic for preventive examinations.

Inclusion Criteria for the Control Group:

- Healthy individuals without multiple sclerosis.
- Males and females aged between 18 and 99 years.

Exclusion Criteria for the Control Group:

- Individuals with subjective neurological complaints.
- Individuals who have undergone spinal anesthesia.
- Individuals with other neurological diseases, excluding those related to demyelinating disorders of the brain and/or spinal cord.

### 2.2. DNA Extraction and Genotyping

After collecting venous blood samples (white blood cells), genomic DNA was extracted using the DNA salting-out method. Genotyping of *TNF-alpha* (rs1800630, rs1800629, and rs361525) was performed using real-time polymerase chain reaction (RT-PCR). The genotyping was conducted on a Step One Plus RT-PCR system (Applied Biosystems, Chicago, IL, USA) with TaqMan^®^ SNP Genotyping Assays (Thermo Scientific, Waltham, MA, USA) following the manufacturer’s protocol.

The specific TaqMan probes and assay IDs used for each polymorphism were:

- rs1800630: TaqMan probe Assay ID C___2215707_10,
- rs1800629: TaqMan probe Assay ID C___7514879_10, and
- rs361525: TaqMan probe Assay ID C___11918230_10.

Genotyping reactions were performed in 96-well plates. Each reaction contained 10 ng of genomic DNA, 5 μL of TaqMan Genotyping Master Mix, 0.25 μL of 20X TaqMan SNP Genotyping Assay, and nuclease-free water to a final volume of 10 μL. The thermal cycling conditions were as follows: initial denaturation at 95 °C for 10 min, followed by 40 cycles of 95 °C for 15 s and 60 °C for 1 min. The Allelic Discrimination program was used during RT-PCR to determine the individual genotypes based on the fluorescence intensity of the different detectors (VIC and FAM).

### 2.3. Statistical Analysis

The statistical analysis for this study was performed using IBM SPSS Statistics 29.0. Initially, descriptive statistics were used to summarize the data. The χ^2^ test and Fisher’s exact test were employed to compare the homogeneity of the genotypes and allele distributions of the *TNF-alpha* gene polymorphisms (rs1800630, rs1800629, and rs361525). Additionally, binary logistic regression was conducted to evaluate the impact of genotypes and alleles on the occurrence of MS. Considering inheritance models and genotype combinations, the odds ratio (OR) was calculated with a 95% confidence interval (CI). The model with the lowest Akaike information criterion (AIC) value was identified as the most suitable inheritance model. The SNPStats program was also used to analyze the haplotypes as part of the analysis.

## 3. Results

A total of 500 subjects took part in this study. They were divided into two groups: a control group (*n* = 250) and a group with MS (*n* = 250). The control group consisted of 85 men (34%) and 165 women (66%), while the MS group consisted of 88 men (35.2%) and 162 women (64.8%). The median age of the control group was 42 years. The median age of the MS group was 39 years. The demographic data are shown in Table 1.

After analyzing the distribution of SNP genotypes and alleles, we found that the AG genotype of the *TNF-alpha* gene rs361525 was statistically significantly less frequent in the MS group than in the control group (4.0% vs. 7.2%, *p* = 0.042) (Table 2). The analysis of the SNP rs1800630 and rs1800629 genotypes and alleles did not yield any statistically significant results (Table 2).

The binary logistic regression analysis with the polymorphisms rs1800630, rs1800629 and rs361525 of the *TNF-alpha* gene yielded no statistically significant results (Table 3).

After analyzing the distribution of genotypes and alleles of the *TNF-alpha* polymorphism rs1800629 in individuals younger than 39 years, it was found that the A allele was statistically significantly less common in the MS group than in the control group (8.6% versus 15.0%, *p* = 0.030) (Table 4). However, rs1800630 and rs631525 polymorphisms analysis yielded no statistically significant results (Table 4).

After performing a binary logistic regression analysis of the *TNF-alpha* gene polymorphism rs1800629, we found that the AA genotype reduced the probability of developing MS by 2 fold compared to the AG + GG genotype (OR = 0.526 (0.282–0.982); *p* = 0.044). And we found that each A allele reduced the probability of developing MS by 2 fold (OR = 0.512 (0.282–0.931); *p* = 0.028). The analysis of the rs1800630 and rs361525 polymorphisms yielded no statistically significant results. The results are shown in Table 5.

The analysis of the distribution of genotypes and alleles of the single-nucleotide polymorphisms of the *TNF-alpha* gene rs1800630, rs1800629, and rs361525 showed no statistically significant results when analyzing subjects aged 39 years and older (Supplementary Material Table S1).

The binary logistic regression analysis with the polymorphisms rs1800630, rs1800629 and rs361525 of the TNF-alpha gene yielded no statistically significant results in the group of people aged 39 years and older (Supplementary Material Table S2).

The analysis of the distribution of genotypes and alleles of the single-nucleotide polymorphisms rs1800630, rs1800629 and rs361525 of the *TNF-alpha* gene in women did not yield statistically significant results (Supplementary Material Table S3).

The binary logistic regression analysis with the polymorphisms rs1800630, rs1800629 and rs361525 of the *TNF-alpha* gene showed no statistically significant results in women with MS and the control group (Supplementary Material Table S4).

After comparing the genotypes and allele distribution of the *TNF-alpha* gene polymorphism rs1800630 in men, we found that the A allele was statistically significantly more common in the MS group than in the control group (21.0% vs. 12.9%, *p* = 0.046). We also found that the distributions of the genotypes of the single-nucleotide polymorphism rs361525 (GG, AG and AA) were statistically significantly different when comparing the MS group with the control group (97.7%, 0% and 2.3% vs. 90.6%, 9.4% and 0%, *p* = 0.005), while the genotype and allele distributions of the rs1800629 polymorphism showed no statistically significant differences (Table 6).

However, we found no statistically significant results after performing a binary logistic regression analysis of the *TNF-alpha* gene polymorphisms rs1800630, rs1800629, and rs361525 in men with MS and the control group (Table 7).

## 4. Discussion

Our study analyzed the single-nucleotide polymorphisms rs1800630, rs1800629 and rs361525 of the *TNF-alpha* gene. Any SNP analysis is very important for the assessment of disease pathogenesis and fundamental analysis, but the scientific reports on TNF-alpha analysis have shown that this is an important clinical aspect of MS. TNF-α also helps to heal tissue and control immune reactions. It is involved in the removal of myelin remnants and the recruitment of regulatory T cells, which help to regulate the immune response and prevent excessive inflammation. TNF-α can also promote remyelination, which is the process of restoring the myelin sheaths around damaged nerve fibers. This means that TNF-α may play a dual role in multiple sclerosis, contributing both to the progression of the disease and to its healing mechanisms [25,26]. MS research aims to reduce the deleterious effects of excessive TNF-αlfa activity while preserving its beneficial effects, but this requires elucidation of the factors that regulate the protein’s expression [24]. The major importance of TNF-α in multiple sclerosis has led to the development of targeted therapies aimed at modulating TNF-α activity. Anti-TNF-α therapies, such as monoclonal antibodies that block TNF-α or its receptors, have been investigated as a treatment for MS. These therapies aim to reduce the harmful effects of excessive TNF-α activity while preserving its beneficial effects. However, the use of anti-TNF-α therapies in multiple sclerosis is still under investigation, and the efficacy and safety continue to be evaluated [26]. Therefore, it is extremely important to find the key regulatory factors of this protein that could open up therapeutic perspectives.

So, no studies could be found in the scientific literature investigating the association between the *TNF-alpha* gene rs1800630 SNP and MS, but there are a number of studies investigating the association between MS and the single-nucleotide polymorphisms rs1800629, rs361525. So far, no statistically significant results have been obtained to confirm that these polymorphisms determine the manifestation of the disease. Tumor necrosis factor-alpha (TNF-α) is an important pro-inflammatory cytokine involved in many processes in our body, e.g., cell differentiation, cell proliferation and cell death. It is also involved in the regulation of inflammation and immune responses. It is known that DNA sequence variations in the promoter region of the *TNF* gene can affect its transcription and thereby influence circulating TNF levels, predisposing and increasing human susceptibility to autoimmune, infectious, oncological and other diseases [22].

In our study, the single-nucleotide polymorphisms rs1800630, rs1800629 and rs361525 of the *TNF-alpha* gene were analyzed. The study sample consisted of 250 healthy volunteers and 250 multiple sclerosis patients.

First, regarding the rs361525 polymorphism, our analysis showed that the AG genotype of the *TNF-alpha* gene rs361525 occurs statistically significantly less frequently in the MS group than in the control group (4.0% vs. 7.2, *p* = 0.042). When analyzing this polymorphism by sex, we found that the distribution of genotypes (GG, AG, and AA) was statistically significantly different in males when comparing the MS group with the control group (97.7%, 0%, and 2.3% vs. 90.6%, 9.4%, and 0%, *p* = 0.005). This suggests that the AG genotype may confer a protective effect against MS in males, or alternatively, that the GG genotype may be more strongly associated with MS in males. This polymorphism is assumed to be one of the most important factors for disease susceptibility in humans, as it can influence the transcription of the *TNF* gene [27]. One study found that patients with the AG genotype produce a lower amount of TNF than patients with the GG genotype, and the GG genotype in MS occurred more frequently [28]. However, these results did not confirm an association between this polymorphism and the occurrence of MS. Nonetheless, statistically significant results confirm that this SNP is associated with the occurrence of other diseases. For example, one study found that individuals with the AA/GA rs361525 genotypes have a lower risk of lung cancer than those with the GG genotype, and the GG genotype accelerates disease progression. Additionally, carriers of the rs361525 GG genotype have a higher risk of developing squamous cell carcinoma of the lung [29].

Regarding the rs1800629 polymorphism, in the group of people younger than 39 years, MS patients with the AA genotype had a reduced odds of developing MS by a factor of two compared to the AG + GG genotype (*p* = 0.044) and each A allele reduced the odds of developing MS by a factor of two (*p* = 0.028). Based on the literature published by other scientists, it was possible to find a link between the rs1800629 polymorphism of the *TNF-alpha* gene and MS. In 2020, Noha M. Bakr and others investigated the tumor necrosis factor-α (*TNF-α) -308 G/A* (rs1800629 G/A) gene polymorphism and its association with MS and clinical signs of the disease in the Egyptian population. Although they did not find statistically significant results demonstrating that the AA genotype and the A allele of the polymorphism *(TNF-α) -308* G/A (rs1800629) were associated with MS susceptibility, they were found to be associated with disease severity and disability progression. The researchers observed a statistically significant increase in Expanded Disability Scale (EDSS) and Multiple Sclerosis Severity Scale (MSFC) mean scores in patients with AA genotype and A allele compared to patients with GG and GA genotype and G allele (*p* < 0.001 and *p* = 0.032), and their regression analysis confirmed that this polymorphism is a predictor of disease severity using the EDSS (*p* = 0.01) [30]. These results can be supported by the observation that in individuals with the *TNF-308* genotype AA/GA, the A allele increases TNF gene expression by 6–7 fold [31].

We found no studies investigating the association of *TNF-863A/C* (rs1800630) SNP with MS. However, in 2019, Ali Yousefian-Jazi and other researchers investigated the influence of this and other SNPs on the manifestation of autoimmune diseases such as atopic dermatitis (AD) and inflammatory bowel disease (IBD). They found that the SNP rs1800630, which is located in the promoter region in the *TNF* gene, is associated with the occurrence of AD and UHL. Their results are based on the fact that the A allele of *TNF-α* rs1800630 has higher promoter activity than the C allele, which leads to increased expression of the *TNF* gene and, at the same time, increases the risk of developing the diseases studied. In their analysis, plasma levels of TNF were found to be significantly higher in both AD and UHL patients than in healthy patients, and the more severe the patient’s condition, the higher the amount of TNF detected in plasma [32]. These observations are consistent with the results of our work with this SNP. When comparing the genotypes and allele distributions of the *TNF-alpha* gene polymorphism rs1800630 in men, we found that the A allele was statistically significantly more frequent in the MS group than in the control group (21.0% vs. 12.9%, *p* = 0.046). This finding suggests that the A allele of rs1800630 may be associated with an increased risk of MS in males. This sex-specific difference highlights the complexity of genetic susceptibility to MS and underscores the importance of considering it in genetic studies of MS. One study was found in the scientific literature that investigated *TNF-863* A/C (rs1800630) SNP with age-related macular degeneration (AMD). It was found that the A allele of *TNF* rs1800630 was more prevalent in AMD patients than in healthy individuals (*p* = 0.029). Recently, inflammatory and immune responses were found to play an important role in the pathogenesis of AMD and MS [33]. Therefore, it can be assumed that *TNF-863* A/C (rs1800630) SNP may influence the manifestation of MS due to impaired *TNF-α* gene expression and increased or decreased circulating TNF-α levels in the body.

Although there are not many papers in the scientific literature investigating and confirming the association between the *TNF-alpha* gene polymorphism rs1800630 and MS, other statistically significant associations have been found between the other *TNF-alpha* gene polymorphisms rs1800629 and rs361525 and this disease. Both in our work and in the studies of other researchers, results were presented showing that the AG genotype of the *TNF-alpha* gene polymorphism rs361525 is statistically significantly less frequent in the group of MS patients and in other diseases in which *TNF* plays an important role. In contrast to the results of our study, a study conducted by other researchers on the rs1800629 polymorphism suggests that the AA genotype and the A allele contribute to higher expression of the *TNF-alpha* gene, which may determine a person’s susceptibility to the disease and its severity. Although there are no other studies investigating the association between the *TNF-alpha* polymorphism rs1800630 and MS, other studies are looking at this polymorphism and its potential impact on similar diseases such as AMD and Alzheimer’s disease. These diseases are considered similar in the context of this research because they also involve chronic inflammatory processes and immune system dysregulation, which are key factors in the pathogenesis of MS. As with our results, the A allele of the *TNF-alpha* gene polymorphism rs1800630 was statistically significantly more common in the groups of diseased individuals than in the healthy individuals. These results highlight the potential importance of these genetic variants in the context of autoimmune and inflammatory diseases. Although our study is observational and does not include functional research, it contributes to a better understanding of the genetic factors that may influence the development and progression of MS. Further research, including functional studies, is necessary to elucidate the mechanisms by which these polymorphisms affect MS and to explore their potential as targets for new treatments.

## 5. Conclusions

In conclusion, our study highlights significant associations between *TNF-alpha* gene variants and MS. Specifically, we observed that the rs631525 AG genotype was less prevalent in the MS group, with notable sex-specific differences. Additionally, the rs1800629 A allele was found to be less frequent in the MS group compared to the control group, and the AA genotype was associated with an approximately 2-fold decrease in the likelihood of MS occurrence compared with the AG + GG genotypes. Furthermore, each A allele of rs1800629 was linked to a 2-fold decreased likelihood of MS occurrence. Interestingly, in males, the rs1800630 A allele was more prevalent in the MS group. These findings underscore the relevance of *TNF-alpha* genetic variations in MS susceptibility, suggesting potential avenues for further research and therapeutic exploration.

## Figures and Tables

**Table 1:** Demographic data.

Characteristic		Group		
		MS Patients, (%)	Control Group (%)	*p*-Value
Sex	Females, *n* (%)	162 (64.8)	165 (66.0)	0.778
	Males, *n* (%)	88 (35.2)	85 (34.0)	
Age median (IQR)		39.0 (15.0)	42.0 (24.0)	0.717 *

* Mann–Whitney U test; IQR—interquartile range.

**Table 2:** Distribution of *TNF-alpha* rs1800630, rs1800629, and rs361525 polymorphism genotypes and allele frequencies between MS and control subjects.

Polymorphism	MS Patients, *n* (%)	Control Group, *n* (%)	HWE *p*-Value	*p*-Value
rs1800630				
CC	182 (72.8)	186 (74.4)	0.001	0.749
AC	51 (20.4)	51 (20.4)		
AA	17 (6.8)	13 (5.2)		
Total	250 (100)	250 (100)		
Allele				
C	415 (83.0)	423 (84.6)		0.492
A	85 (17.0)	77 (15.4)		
rs1800629				
GG	202 (80.8)	186 (74.4)	0.417	0.227
AG	46 (18.4)	61 (24.4)		
AA	2 (0.8)	3 (1.2)		
Total	250 (100)	250 (100)		
Allele				
G	450 (90.0)	433 (86.6)		0.094
A	50 (10.0)	67 (13.4)		
rs361525				
GG	236 (94.4)	232 (92.8)	0.555	0.042
AG	10 (4.0)	18 (7.2)		
AA	4 (1.6)	0 (0)		
Total	250 (100)	250 (100)		
Allele				
G	482 (96.4)	482 (96.4)		1.00
A	18 (3.6)	18 (3.6)		

**Table 3:** Binary logistic regression analysis of *TNF-alpha* rs1800630, rs1800629, and rs361525 between MS patients and controls.

Model	Genotype/Allele	OR (95% CI)	*p*-Value	AIC
*rs1800630*				
Codominant	AC vs. AA	1.022 (0.659–1.585)	0.923	696.569
	CC vs. AA	1.336 (0.631–2.831)	0.449	
Dominant	AC + CC vs. AA	1.086 (0.729–1.616)	0.685	694.982
Recessive	CC vs. AA + AC	1.330 (0.632–2.800)	0.453	694.578
Overdominant	AC vs. AA + CC	1.000 (0.647–1.545)	1.000	695.147
Additive	A	1.099 (0.813–1.486)	0.539	694.769
*rs1800629*				
Codominant	AG vs. AA	0.694 (0.451–1.069)	0.098	694.176
	GG vs. AA	0.614 (0.101–3.715)	0.595	
Dominant	AG + GG vs. AA	0.691 (0.452–1.055)	0.087	692.194
Recessive	GG vs. AA + AG	0.664 (0.110–4.008)	0.655	694.944
Overdominant	AG vs. AA + GG	0.699 (0.454–1.075)	0.103	690.465
Additive	A	0.709 (0.476–1.055)	0.090	692.234
*rs361525*				
Codominant	AG vs. AA	0.546 (0.247–1.208)	0.135	689.250
	GG vs. AA	-	-	
Dominant	AG + GG vs. AA	0.765 (0.372–1.573)	0.466	694.612
Recessive	GG vs. AA + AG	-	0.999	689.570
Overdominant	AG vs. AA + GG	0.537 (0.243–1.188)	0.125	692.694
Additive	A	1.000 (0.544–1.839)	1.000	695.147

**Table 4:** Distribution of genotypes and allele frequencies of the *TNF-alpha* polymorphism rs1800630, rs1800629, and rs361525 in individuals younger than 39 years of age.

Polymorphism	MS Patients, *n* (%)	Control Group, *n* (%)	*p*-Value
rs1800630			
CC	84 (68.9)	82 (72.6)	0.271
AC	23 (18.9)	24 (21.2)	
AA	15 (12.2)	7 (6.2)	
Total	122 (100)	113 (100)	
Allele			
C	191 (85.2)	188 (83.3)	0.179
A	53 (14.8)	38 (16.7)	
rs1800629			
GG	101 (82.8)	81 (71.7)	0.066
AG	21 (17.2)	30 (26.4)	
AA	0 (0)	2 (1.9)	
Total	122 (100)	113 (100)	
Allele			
G	223 (91.4)	192 (85.0)	0.030
A	21 (8.6)	34 (15.0)	
rs361525			
GG	114 (93.4)	106 (93.8)	0.363
AG	6 (4.9)	7 (6.2)	
AA	2 (1.7)	0 (0)	
Total	122 (100)	113 (100)	
Allele			
G	234 (95.9)	219 (96.9)	0.561
A	10 (4.1)	7 (3.1)	

**Table 5:** Binary logistic regression analysis of *TNF-alpha* rs1800630, rs1800629, and rs361525 between younger than 39 years of age MS patients and controls group subjects.

Model	Genotype/Allele	OR (95% CI)	*p*-Value	AIC
*rs1800630*				
Codominant	AC vs. AA	0.936 (0.489–1.788)	0.840	326.757
	CC vs. AA	2.092 (0.811–5.395)	0.127	
Dominant	AC + CC vs. AA	1.197 (0.681–2.102)	0.532	327.044
Recessive	CC vs. AA + AC	2.123 (0.832–5.415)	0.115	324.798
Overdominant	AC vs. AA + CC	0.862 (0.454–1.633)	0.648	327.226
Additive	A	1.263 (0.848–1.882)	0.250	326.095
*rs1800629*				
Codominant	AG vs. AA	0.561 (0.299–1.054)	0.072	324.208
	GG vs. AA	-	-	
Dominant	AG + GG vs. AA	0.526 (0.282–0.982)	0.044	323.277
Recessive	GG vs. AA + AG	-	-	324.487
Overdominant	AG vs. AA + GG	0.575 (0.307–1.079)	0.085	324.419
Additive	A	0.512 (0.282–0.931)	0.028	322.436
*rs361525*				
Codominant	AG vs. AA	0.797 (0.26–2.448)	0.692	326.639
	GG vs. AA	-	-	
Dominant	AG + GG vs. AA	1.063 (0.373–3.031)	0.910	327.421
Recessive	GG vs. AA + AG	-	-	324.796
Overdominant	AG vs. AA + GG	0.783 (0.255–2.405)	0.669	327.252
Additive	A	1.273 (0.518–3.129)	0.599	327.153

**Table 6:** Distribution of genotypes and allele frequencies of the *TNF-alpha* rs1800630, rs1800629, and rs361525 polymorphism in men.

Polymorphism	MS Patients, *n* (%)	Control Group, *n* (%)	*p*-Value
rs1800630			
CC	60 (68.2)	66 (77.6)	0.174
AC	19 (21.6)	16 (18.8)	
AA	9 (10.2)	3 (3.5)	
Total	88 (100)	85 (100)	
Allele			
C	139 (79.0)	148 (87.1)	0.046
A	37 (21.0)	22 (12.9)	
rs1800629			
GG	72 (81.8)	64 (75.3)	0.262
AG	16 (18.2)	19 (22.4)	
AA	0 (0)	2 (2.4)	
Total	88 (100)	85 (100)	
Allele			
G	160 (90.9)	147 (86.5)	0.192
A	16 (9.1)	23 (13.5)	
rs361525			
GG	86 (97.7)	77 (90.6)	0.005
AG	0 (0)	8 (9.4)	
AA	2 (2.3)	0 (0)	
Total	88 (100)	85 (100)	
Allele			
G	172 (97.7)	162 (95.3)	0.216
A	4 (2.3)	8 (4.7)	

**Table 7:** Binary logistic regression analysis of *TNF-alpha* rs1800630, rs1800629, and rs361525 between MS and control group males.

Model	Genotype/Allele	OR (95% CI)	*p*-Value	AIC
*rs1800630*				
Codominant	AC vs. AA	1.306 (0.616–2.769)	0.486	240.146
	CC vs. AA	3.300 (0.853–12.763)	0.084	
Dominant	AC + CC vs. AA	1.621 (0.822–3.198)	0.163	239.809
Recessive	CC vs. AA + AC	3.114 (0.813–11.924	0.097	238.634
Overdominant	AC vs. AA + CC	1.187 (0.564–2.499)	0.651	241.571
Additive	A	1.582 (0.944–2.651)	0.082	238.620
*rs1800629*				
Codominant	AG vs. AA	0.749 (0.355–1.577)	0.446	240.328
	GG vs. AA	-	-	
Dominant	AG + GG vs. AA	0.677 (0.326–1.409)	0.297	240.680
Recessive	GG vs. AA + AG	-	-	238.910
Overdominant	AG vs. AA + GG	0.772 (0.367–1.625)	0.495	241.311
Additive	A	0.635 (0.320–1.258)	0.193	240.043
*rs361525*				
Codominant	AG vs. AA GG vs. AA	-	-	229.469
Dominant	AG + GG vs. AA	0.224 (0.046–1.086)	0.063	237.477
Recessive	GG vs. AA + AG	-	-	239.050
Overdominant	AG vs. AA + GG	-	-	230.005
Additive	A	0.557 (0.187–1.662)	0.294	240.579

## Data Availability

Data is available upon request.

## Supplementary Materials

The following supporting information can be downloaded at: https://www.mdpi.com/article/10.3390/jcm13133693/s1. Table S1. Genotype and allele frequency distribution of the *TNF-alpha* polymorphism rs1800630, rs1800629, rs361525 in subjects aged 39 years and older. Table S2. Binary logistic regression analysis of *TNF-alpha* rs1800630, rs1800629, rs361525 between MS patients and control group subjects aged 39 years and older. Table S3. Distribution of genotypes and allele frequencies of the *TNF-alpha* rs1800630, rs1800629, rs361525 polymorphism in females. Table S4. Binary logistic regression analysis of *TNF-alpha* rs1800630, rs1800629, rs361525 between MS group and control group females.
