# Supplementary material for: TNF-alfa Gene Polymorphism Associations with Multiple Sclerosis
Source: J Clin Med. 2024 Jun 25;13(13):3693. doi: 10.3390/jcm13133693 (PMC11242879; doi:10.3390/jcm13133693)
Supplement: Supplementary file 1 [file jcm-13-03693-s001.zip › jcm-3017882-supplementary.pdf]

Supplementary material

Table S1. Genotype and allele frequency distribution of the TNF-alpha polymorphism rs1800630, rs1800629, rs361525 in subjects aged 39 years and older.

**Polymorphism**	**MS patients, N (%)**	**Control group, N (%)**	**p-value**
**rs1800630**			
**CC**	98 (76.7)	104 (75.9)	0.388
**AC**	28 (21.9)	27 (19.7)	
**AA**	2 (1.6)	6 (4.4)	
**Total**	128 (100)	137 (100)	
**Allele**			
**C**	224 (87.5)	235 (85.8)	0.558
**A**	32 (12.5)	39 (14.2)	
**rs1800629**			
**GG**	101 (78.9)	105 (76.6)	0.688
**AG**	25 (19.5)	31 (22.6)	
**AA**	2 (1.6)	1 (0.7)	
**Total**	128 (100)	137 (100)	
**Allele**			
**G**	227 (88.7)	241 (88.0)	0.798
**A**	29 (11.3)	33 (12.0)	
**rs361525**			
**GG**	122 (95.3)	126 (92.0)	0.081
**AG**	4 (3.1)	11 (8.0)	
**AA**	2 (1.6)	0 (0)	
**Total**	128 (100)	137 (100)	
**Allele**			
**G**	248 (96.9)	263 (96.0)	0.582
**A**	8 (3.1)	11 (4.0)	

Table S2. Binary logistic regression analysis of TNF-alpha rs1800630, rs1800629, rs361525 between MS patients and control group subjects aged 39 years and older.

Model	Genotype/allele	OR (95% CI)	*P-value*	AIC
		**rs1800630**		
**Codominant**	**AC *vs.* AA**<br>**CC *vs.* AA**	1.101 (0.606-1998)<br>0.354 (0.070-1.795)	0.753<br>0.210	369.079
**Dominant**	**AC+CC *vs.* AA**	0.965 (0.548-1.699)	0.901	369.047
**Recessive**	**CC *vs.* AA+AC**	0.8347 (0.069-1.749)	0.200	367.178
**Overdominant**	**AC *vs.* AA+CC**	1.141 (0.630-2.066)	0.664	368.873
**Additive**	**A**	0.873 (0.541 -1.408)	0.578	368.751
		**rs1800629**		

**Codominant**	**AG *vs.* AA**<br>**GG *vs.* AA**	0.838(0.463-1.518)<br>2.079 (0.186-23.288)	0.560<br>0.553	370.306
**Dominant**	**AG+GG *vs.* AA**	0.877 (0.491-1.567)	0.658	368.866
**Recessive**	**GG *vs.* AA+AG**	2.159 (0.193-24.099)	0.532	368.646
**Overdominant**	**AG *vs.* AA+GG**	0.830 (0.459-1.501)	0.538	368.681
**Additive**	**A**	0.931 (0.544-1.594)	0.796	368.995
		**rs361525**		
**Codominant**	**AG *vs.* AA**<br>**GG *vs.* AA**	0.376 (0.116-1.211)<br>-	0.101<br>-	365.134
**Dominant**	**AG+GG *vs.* AA**	0.563 (0.202-1.571)	0.736	367.811
**Recessive**	**GG *vs.* AA+AG**	-	-	366.135
**Overdominant**	**AG *vs.* AA+GG**	0.370 (0.115-1.192)	0.096	365.955
**Additive**	**A**	0.802 (0.341-1.888)	0.614	368.804

Table S3. Distribution of genotypes and allele frequencies of the *TNF-alpha* rs1800630, rs1800629, rs361525 polymorphism in females.

**Polymorphism**	**MS patients, N (%)**	**Control group, N (%)**	**p-value**
**rs1800630**			
**CC**	122 (75.3)	120 (72.7)	0.841
**AC**	32 (19.8)	35 (21.2)	
**AA**	8 (4.9)	10 (6.1)	
**Total**	162 (100)	165 (100)	
**Allele**			
**C**	276 (85.2)	275 (83.3)	0.516
**A**	48 (14.8)	55 (16.7)	
**rs1800629**			
**GG**	130 (80.2)	122 (73.9)	0.278
**AG**	30 (18.5)	42 (25.5)	
**AA**	2 (1.2)	1 (0.6)	
**Total**	162 (100)	165 (100)	
**Allele**			
**G**	290 (89.5)	286 (86.7)	0.263
**A**	34 (10.5)	44 (13.3)	
**rs361525**			
**GG**	150 (92.6)	155 (93.9)	0.358
**AG**	10 (6.2)	10 (6.1)	
**AA**	2 (1.2)	0 (0)	
**Total**	162 (100)	165 (100)	
**Allele**			
**G**	310 (95.7)	320 (97.0)	0.380
**A**	14 (4.3)	10 (3.0)	

Table S4. Binary logistic regression analysis of *TNF-alpha* rs1800630, rs1800629, rs361525 between MS group and control group females.

Model	Genotype/allele	OR (95% CI)	*P value*	AIC
	**rs1800630**			
**Codominant**	**AC *vs.* AA**<br>**CC *vs.* AA**	0.899 (0.523-1.546)<br>0.787 (0.300-2.062)	0.701<br>0.626	456.945
**Dominant**	**AC+CC *vs.* AA**	0.874 (0.533-1.434)	0.595	455.007
**Recessive**	**CC *vs.* AA+AC**	0.805 (0.805-2.095)	0.657	455.092
**Overdominant**	**AC *vs.* AA+CC**	0.914 (0.534-1.565)	0.744	455.184
**Additive**	**A**	0.892 (0.610 -1.306)	0.558	454.946
	**rs1800629**			
**Codominant**	**AG *vs.* AA**<br>**GG *vs.* AA**	0.670 (0.395-1.138)<br>1.877 (0.168-20.963)	0.139<br>0.609	454.715
**Dominant**	**AG+GG *vs.* AA**	0.698 (0.415-1.175)	0.176	453.445
**Recessive**	**GG *vs.* AA+AG**	2.050 (0.184-22.832)	0.559	454.929
**Overdominant**	**AG *vs.* AA+GG**	0.666 (0.392-1.130)	0.131	452.991
**Additive**	**A**	0.751 (0.461-1.226)	0.252	453.968
	**rs361525**			
**Codominant**	**AG *vs.* AA**<br>**GG *vs.* AA**	1.033 (0.418-2.554)<br>-	0.943<br>-	454.464<br>-
**Dominant**	**AG+GG *vs.* AA**	1.240 (0.520-2.956)	0.627	455.054
**Recessive**	**GG *vs.* AA+AG**	-	-	-
**Overdominant**	**AG *vs.* AA+GG**	1.020 (0.413-2.520)	0.966	455.289
**Additive**	**A**	1.385 (0.635-3.022)	0.413	454.608
